# Protein tyrosine phosphatase receptor delta acts as a neuroblastoma tumor suppressor by destabilizing the aurora kinase a oncogene

**DOI:** 10.1186/1476-4598-11-6

**Published:** 2012-02-05

**Authors:** Maria Meehan, Laavanya Parthasarathi, Niamh Moran, Caroline A Jefferies, Niamh Foley, Elisa Lazzari, Derek Murphy, Jacqueline Ryan, Berenice Ortiz, Armida W M Fabius, Timothy A Chan, Raymond L Stallings

**Affiliations:** 1 Department of Molecular and Cellular Therapeutics, Royal College of Surgeons in Ireland, Dublin 2, Dublin, Ireland; 2 National Children's Research Centre, Our Lady's Children's Hospital, Dublin 12, Dublin, Ireland; 3 Centre for Human Proteomics, Royal College of Surgeons in Ireland, Dublin 2, Dublin, Ireland; 4 Department of Radiation Oncology, Memorial Sloan-Kettering Cancer Center, 1275 York Avenue, New York, NY 10065, USA

**Keywords:** PTPRD, AURKA, MYCN, Neuroblastoma, Tumor suppressor

## Abstract

**Background:**

Protein tyrosine phosphatase receptor delta (PTPRD) is a member of a large family of protein tyrosine phosphatases which negatively regulate tyrosine phosphorylation. Neuroblastoma is a major childhood cancer arising from precursor cells of the sympathetic nervous system which is known to acquire deletions and alterations in the expression patterns of *PTPRD*, indicating a potential tumor suppressor function for this gene. The molecular mechanism, however, by which PTPRD renders a tumor suppressor effect in neuroblastoma is unknown.

**Results:**

As a molecular mechanism, we demonstrate that PTPRD interacts with aurora kinase A (AURKA), an oncogenic protein that is over-expressed in multiple forms of cancer, including neuroblastoma. Ectopic up-regulation of PTPRD in neuroblastoma dephosphorylates tyrosine residues in AURKA resulting in a destabilization of this protein culminating in interfering with one of AURKA's primary functions in neuroblastoma, the stabilization of MYCN protein, the gene of which is amplified in approximately 25 to 30% of high risk neuroblastoma.

**Conclusions:**

PTPRD has a tumor suppressor function in neuroblastoma through AURKA dephosphorylation and destabilization and a downstream destabilization of MYCN protein, representing a novel mechanism for the function of PTPRD in neuroblastoma.

## Background

Protein tyrosine phosphatase receptor delta (*PTPRD*) is an important regulator of axon growth and guidance and is highly expressed in the central nervous system where it functions as a transmembrane homophilic neuronal cell adhesion molecule [1]. *PTPRD *undergoes a high frequency of hemizygous/homozygous deletions in multiple forms of cancer, which are often intragenic in nature, indicating a potential tumor suppressor function [2-8]. Additional mechanisms leading to PTPRD inactivation include promoter region hypermethylation, point mutations and aberrant splicing [6,9-12].

Neuroblastoma is derived from primitive cells of the sympathetic nervous system, and is the most common extracranial solid tumor in children accounting for 15% of all childhood cancer deaths [13]. These tumors are particularly noted for extensive heterogeneity in clinical behaviour, ranging from spontaneous regression to aggressive clinical course and death from disease. Notably, amplification of the *MYCN *transcription factor is one of the most powerful adverse prognostic factors in neuroblastoma [14] and we have previously demonstrated that *PTPRD *is expressed at significantly lower levels in *MYCN *amplified neuroblastoma relative to non-*MYCN *amplified tumors [10]. In addition, *PTPRD *mRNA expression is higher in normal adrenal fetal neuroblasts, the cell of origin of neuroblastoma, relative to unfavourable neuroblastoma tumors, indicating that *PTPRD *down-regulation might be an important step in the development of these tumors [7,10]. Multiple mechanisms appear to exist for the down-regulation of *PTPRD *in neuroblastoma, including intragenic microdeletions which can include coding sequence, or in some instances be restricted to non-coding exons of an extended 5' UTR [5]. Aberrant splicing of the 5' UTR also has been noted in neuroblastoma cell lines and primary tumors, which could potentially cause destabilization of the mRNA sequence [10].

In this report, we demonstrate for the first time that experimental up-regulation of *PTPRD *in neuroblastoma cell lines significantly decreases cell growth and increases apoptosis. Moreover, we identify aurora kinase A, a serine/threonine kinase oncogene that is up-regulated in many forms of cancer, including high risk neuroblastoma [15], as an interaction partner of PTPRD. We further demonstrate that PTPRD has a tumor suppressor function in neuroblastoma through dephosphorylating and destabilizing AURKA, leading to a downstream decrease of MYCN protein. Our findings represent a novel mechanism of action for the function of PTPRD in neuroblastoma.

## Results

### PTPRD functions as a tumor suppressor in neuroblastoma

**Figure 1:**
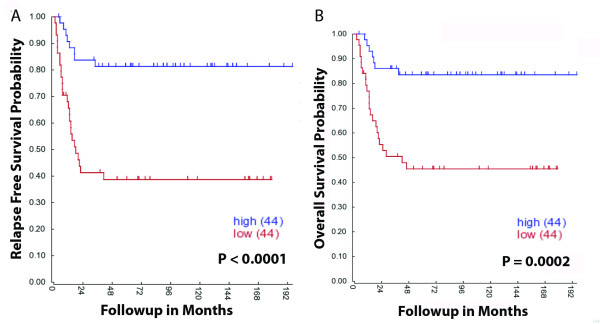
**Kaplan Meier survival curves demonstrating the relationship between patient survival and PTPRD gene expression using the R2: microarray analysis and visualization platform (http://r2.amc.nl)**. (A) is relapse free survival and (B) is overall survival where low expression is < median (n = 44 (tumors) and high expression is > median (n = 44 tumors).

In order to further examine the possibility that *PTPRD *acts as a tumor suppressor gene in neuroblastoma, we initially analyzed the levels of *PTPRD *mRNA transcripts in a set of 88 neuroblastoma tumors using the R2: microarray analysis and visualization platform (http://r2.amc.nl) (University of Amsterdam). Lower than median *PTPRD *mRNA levels were significantly associated with both poor relapse free and overall patient survival, consistent with *PTPRD *having a tumor suppressor function (Figure 1A and 1B).

**Figure 2:**
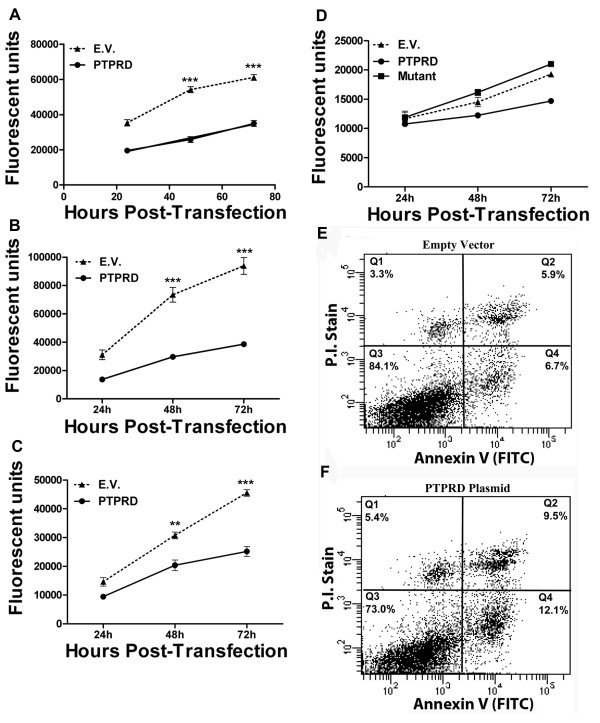
**Ectopic over-expression of wild type PTPRD suppresses the growth of human neuroblastoma cell lines by inducing cell apoptosis**. (**A**) SHSY5Y (**B**) Kelly and (**C**) CHP-212 cells were transfected with PTPRD cDNA or empty vector (pcDNA3.1-V5) and were analysed by a Cyquant assay at 24, 48 and 72 h. All graphs report mean ± S.E.M., n = 3, ** *p *< 0.01, *** *p *< 0.001. (**D**) Kelly cells transfected with either empty vector or an expression plasmid containing PTPRD with a mutated phosphatase domain. PTPRD induced apoptosis was demonstrated by FACS analysis of annexin-V staining. Kelly cells were transfected with empty vector (**E**) or PTPRD cDNA (**F**). Cells were harvested 72 h post transfection. PI staining of DNA represents necrotic cells (upper left quadrant). Annexin V staining indicates cells that are early apoptotic (bottom right quadrant). Double stained cells (upper right quadrant) indicate cells in late apoptosis. Percentages of cells in each quadrant are shown.

We then sought to determine the effects of *PTPRD *over-expression in neuroblastoma cell lines that have only minimally detectable *PTPRD *mRNA transcripts. Kelly cells (*MYCN *amplified) have a homozygous deletion in the *PTPRD *5' UTR [5], while SHSY-5Y (*MYCN *non-amplified) has a 5' UTR that was generated by exon skipping [10]. Although there is no evidence for deletion or aberrant splicing of *PTPRD *in CHP212 (*MYCN *amplified), endogenous mRNA transcripts are nevertheless at very low levels. These three cell lines were transfected with a wild type *PTPRD *V5-tagged cDNA clone [6] to determine the impact of over-expression on cell growth. Ectopic expression of *PTPRD *at mRNA and protein level was validated by TaqMan qPCR and by Western blot analysis (for a V5 epitope tag) (Additional File 1). Ectopic up-regulation of PTPRD resulted in a significant decline in cell growth in all three cell lines (Figure 2A-C). To determine if the phosphatase activity of PTPRD is required to have anti-proliferative effects on cell lines, a mutant form of *PTPRD *containing a cancer specific mutation, Q1481X, that results in a truncated protein product lacking a functional C-terminal phosphatase domain, was transfected into Kelly cells. Over-expression of this *PTPRD *mutant did not lead to a significant decrease in cell growth compared to empty vector transfected cells (Figure 2D), indicating that a functional phosphatase domain is required. FACS analysis of Kelly cells transfected with either wild type PTPRD or empty vector, followed by staining with propidium iodide and staining for Annexin V, revealed that *PTPRD *up-regulation resulted in a median 1.7 fold increase in apoptosis relative to empty vector control (Figure 2E, F).

### Identification of proteins that interact with PTPRD

Taken together, all available evidence indicates that PTPRD is a neuroblastoma tumor suppressor, but the molecular mechanism(s) is unknown, as only a small number of PTPRD interacting partners have been identified. PTPRD is known to interact with the metastasis suppressor protein 1 (MTSS1), liprin-alpha 1 (LAR), and signal transducer and activator of transcription 3 (STAT3) [6,16,17]. In order to discover new potential substrates for PTPRD in the context of neuroblastoma, we used a commercially available protein-protein interaction array containing 9,400 proteins. A biotinylated PTPRD protein was used to probe the protein microarray to detect potential interaction partners. This experiment was conducted in duplicate (biological replicates as two different biotinylated PTPRD baits were used). The consensus interacting proteins with above-background signal are listed in Table 1.

**Table 1:** Protein Interaction Array Results

Gene ID	Array ID	Z-score 1A*	Z-score 1B	Z-score 2A	Z-score 2B	Mean Score
**SLAIN2**	BC031691.2	15.6862	14.84417	26.53102	26.53143	20.898205

**AURKA**	PV3612	17.81632	17.54567	23.21736	21.82452	20.1009675

**NEK1**	PV4202	11.32571	10.6591	26.53388	26.53388	18.7631425

**PLK1**	PV3501	10.43356	7.98767	26.28537	24.45825	17.2912125

**EPHA8**	PV3844	13.1952	11.49612	21.98299	21.87788	17.1380475

**TBK1**	PV3504	12.278	11.42595	21.61471	18.89013	16.0521975

**CSNK1D**	PV3665	6.59933	4.8401	23.86103	21.60616	14.226655

**CSNK1E**	PV3500	4.19855	3.59711	15.00776	14.78818	9.3979

**MARK2**	PV3878	5.18092	4.76492	11.28101	13.46825	8.673775

**STK22B**	PV3622	4.70477	4.58448	9.74924	10.31917	7.339415

**SMTNL2**	NM_198501.1	8.20319	7.9576	6.70486	6.47999	7.33641

**PAK6**	PV3502	6.12319	4.86015	9.65432	8.0142	7.162965

**NEK2**	PV3360	3.79759	3.28135	10.46746	10.817	7.09085

**DDX17**	NM_030881.2	11.17033	10.22806	3.26491	3.22825	6.9728875

**NEK6**	PV3353	7.72705	7.69697	5.83062	5.50919	6.6909575

**ABLIM1**	BC002448.2	7.49649	4.08829	7.04747	7.37379	6.50151

**SCEL**	BC020726.1	4.70978	2.7651	10.60149	5.45623	5.88315

**BMX**	PV3371	5.37138	5.07566	5.66807	4.98611	5.275305

**MATK**	PV3370	3.42168	3.27633	6.37244	7.68829	5.189685

**IRS1**	BC053895	5.80241	5.78237	3.98394	2.59843	4.5417875

**DDX54**	BC001132.1	3.27132	2.99065	3.27469	3.16062	3.17432

*1A and 1B are technical replicates using the same protein bait. 2A and 2B are technical replicates using a second bait are therefore biological replicates of 1A and 1B.

**Figure 3:**
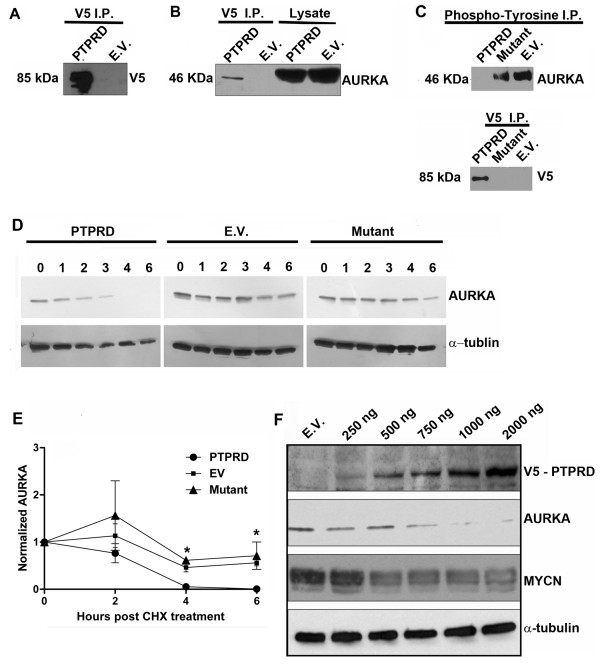
**V5 immunoprecipitation experiments and western blots**. (**A**) Kelly cells were transfected with PTPRD cDNA or empty vector (pcDNA3.1-V5). V5 Immunoprecipitates were subjected to western blot with an AURKA antibody in order to confirm *in vivo *association of AURKA and PTPRD. (**B**) Western blot of the same immunoprecipitates with a V5 antibody demonstrates the pull down of V5 PTPRD. (**C**) Kelly cells were transfected with PTPRD cDNA, empty vector or PTPRD mutant (Q1481X). Post pan-phosphotyrosine immunoprecipitation (pY) and western blotting with AURKA reveals the phosphorylation status of AURKA in these transfectants. Immunoprecipitation of the V5 tag and subsequent western blotting with V5 confirms upregulation of V5-PTPRD (**D) **Ectopic up-regulation of PTPRD results in destabilization of AURKA. Kelly cells were transfected with PTPRD cDNA, empty vector or the phosphatase dead PTPRD mutant Q14811. Lysates were harvested 72 hours post transfection and treated at 0, 1, 2, 3, 4 and 6 hours with cycloheximide and then subjected to western blot analysis with an AURKA antibody. Alpha tubulin was used as a loading control. (**E**) The graph shows quantitative densitometry of the protein expression of AURKA (n = 2) following normalization with the endogenous control. (**F**) Ectopic up-regulation of PTPRD results in a decrease in protein levels of AURKA. and MYCN. Increasing concentrations of PTPRD cDNA were transfected into Kelly cells. Lysates were harvested 72 hours post transfection and subjected to western blot analysis with V5, AURKA and MYCN antibodies. Alpha tubulin was used as a loading control

One of the top scoring proteins on the PTPRD interaction arrays was aurora kinase A (AURKA), a serine/threonine kinase that is involved in microtubule formation and stabilization at the spindle pole during chromosome segregation [18]. In neuroblastoma, high AURKA expression is associated with poor patient survival [15], so that the interaction of a tumor suppressor with AURKA was of immediate interest. In order to confirm an *in vivo *interaction between PTPRD and AURKA in neuroblastoma, protein extract isolated from Kelly cells transfected with either the V5-tagged PTPRD cDNA or empty vector was immunoprecipitated with a V5 epitope antibody. As illustrated in Figure 3A, western blotting demonstrated that V5-tagged PTPRD was immunoprecipitated from cells, with a clear band of approximately 85 kDa being detected compared with empty vector control. The PTPRD pro-protein (220 kDa) is cleaved intracellularly into two subunits of 150 kDa (E-subunit) and 85 kDa (P-subunit) [19]. This process is called "ectodomain shedding" and affects a number of transmembrane proteins. The 150-kDa E-subunit is predicted to contain the three Ig-like and eight FN-III-like domains, whereas the 85-kDa P-subunit contains a short ectodomain segment, the transmembrane peptide, and the intracellular PTPase domains and is expressed in the membrane of the cell. The 150 kDa band is not visualized because it has lost the V5 epitope tag. Western blot probed with AURKA antibody indicates that AURKA co-immunoprecipitates with the V5 epitope pulled down with the V5 antibody, confirming a PTPRD-AURKA interaction *in vivo *(Figure 3B).

### PTPRD destabilizes AURKA by dephosphorylating tyrosine sites

Having confirmed a PTPRD-AURKA interaction, we next investigated whether AURKA is a potential substrate for PTPRD. Within the catalytic domain of AURKA, there are a number of conserved tyrosine phosphorylation sites (Y148, Y199, Y197, Y212) [20]). Immunoprecipitation of tyrosine phosphorylated proteins with a pan-phosphotyrosine agarose conjugate and subsequent western blotting with an AURKA antibody revealed an abolishment of AURKA tyrosine phosphorylation in PTPRD cDNA transfected cells compared to phosphatase dead mutant or empty vector control transfectants, confirming the mechanism of action of PTPRD (Figure 3C). In addition to deactivating AURKA, ectopic over-expression of PTPRD also leads to a decrease in protein stability of AURKA. After 4 hours of cycloheximide treatment, there is a significant difference in the decay of AURKA in the PTPRD transfected cells compared to empty vector (EV) or phosphatase dead mutant (Figure 3D, E). qPCR analysis of AURKA mRNA at 48 hours post PTPRD transfection indicated no significant change relative to negative control, confirming that the AURKA decrease is post-transcriptional (Additional File 2A).

A critical function of AURKA in neuroblastoma is to regulate MYCN protein stability by sequestering MYCN from ubiquitin-mediated proteolytic degradation mediated by the FBXW7 ubiquitin ligase [21], leading us to hypothesize that a down-stream effect of ectopic over-expression PTPRD in neuroblastoma cells would be the destabilization of MYCN. As illustrated in Figure 3F, MYCN protein levels are also decreasing as a consequence of PTPRD ectopic over-expression. The decrease in MYCN protein is due to a post-transcriptional mechanism, as there is no change in MYCN mRNA (Additional File 2B).

## Discussion

In this report we demonstrate a novel *in vivo *interaction between the tumor suppressor PTPRD and the oncogenic AURKA protein in neuroblastoma. Furthermore, to our best knowledge, this is the first report to demonstrate dephosphorylation of tyrosine residues in AURKA leading to its subsequent destabilization. The identification of AURKA as a novel target of PTPRD mediated dephosphorylation adds to a growing list of substrates for this tumor suppressor. Activity of AURKA is also regulated by phosphorylation of Thr288 in a cell cycle dependent manner [22]. Constitutive phosphorylation on Ser51 of AURKA has also been shown to be associated with its over-expression and protein stabilization, preventing protein degradation mediated by the ubiquitin-proteasome system [23] and it is likely that constitutive phosphorylation of tyrosine residues in AURKA also prevent its degradation. AURKA destabilization also has the down-stream effect of destabilizing MYCN, consistent with prior findings by Otto et al. [21]. Thus, PTPRD is likely to have a tumor suppressor function in any type of cancer with MYCN amplification, including other pediatric cancers such as medulloblastoma and rhabdomyosarcoma, or in adult tumors that are dependent on high AURKA levels.

MYCN directly up-regulates AURKA by binding to its promoter region, forming an auto regulatory feedback loop [21]. As previously mentioned, PTPRD expression is significantly lower in MYCN amplified tumors relative to non-MYCN amplified tumors [10], indicating that MYCN might be directly repressing PTPRD in order to maintain AURKA levels. However, analysis of a previously published data set on MYCN binding sites in neuroblastoma cell lines indicated that MYCN does not bind to the PTPRD promoter region [24,25], revealing that down-regulation of *PTPRD *in *MYCN *amplified tumors is an indirect effect.

Along with AURKA, a number of other proteins scored highly in our protein-protein interaction arrays, including SLAIN2, and two serine/threonine kinases, NEK2 and PLK1.

SLAIN2 has recently been shown to play an important role in the microtubule complex by controlling microtubule dynamics and organization [26]. NEK2 is an integral component of the mitotic spindle-assembly checkpoint which is necessary for proper chromosome segregation during metaphase-anaphase transition [27], while PLK1 is an early trigger for G2/M transition [28]. Intriguingly, one of the primary functions of AURKA is the activation of PLK1 by direct phosphorylation of Thr210 [29], and high expression of PLK1 is also significantly associated with high-risk neuroblastoma and unfavourable patient outcome [30]. Inhibition of PLK1 protein has an anti-proliferative effect on neuroblastoma cell proliferation [30]. Although the interaction of PTPRD with these proteins requires validation, it seems likely that over-expression of PTPRD should have a pleiotrophic tumor suppressor effect by associating with, and dephosphorylating a number of interacting partners. The recent development of AURKA inhibitors, along with impressive results in pre-clinical models of pediatric cancer, indicate that AURKA could be a therapeutic target of great value in the treatment of neuroblastoma [31,32]. PTPRD, as a natural antagonist of AURKA, might be of therapeutic value in cancers where its inactivation is epigentically reversible, such as in instances of promoter region hypermethylation.

## Conclusions

Over-expression of PTPRD in neuroblastoma cell lines results in decreased cell viability through the activation of apoptosis. We further demonstrate a novel PTPRD-AURKA protein interaction, and that PTPRD over-expression results in the dephosphorylation and the destabilization of AURKA, along with a down-stream destabilization of MYCN protein. These findings, taken together, indicate a novel mechanism of action for PTPRD as a neuroblastoma tumor suppressor through the inhibition of these important oncogene products.

## Methods

### Cell Culture, *In Vitro *Growth and Apoptosis Assays

SHSY-5Y and CHP-212 neuroblastoma cell lines were obtained from the American Type Culture Collection, while Kelly was obtained from European Collection of Animal Cell Cultures. All cell lines were validated for the presence of previously published genomic imbalances using aCGH. Culture media was supplemented with 10% FBS.

Cells were transfected with 2 μg PTPRD cDNA, empty vector (pcDNA3.1-V5) or phosphatase dead mutant PTPRD (Q14811) using lipofectamine 2000 reagent according to

the manufacturer's instructions (Invitrogen, Carlsbad, CA) and were analysed by a Cyquant assay (Invitrogen) at 24, 48 and 72 h. PTPRD induced apoptosis was demonstrated by FACS analysis of annexin-V staining. Kelly cells were transfected with empty vector or PTPRD cDNA. Cells were harvested 72 h post transfection and apoptosis was quantified using the FITC Annexin-V Apoptosis Detection Kit I (BD Pharmingen, San Diego, CA, USA). Experiments were performed in triplicate to quantify apoptosis by phosphatidylserine (PS) externalization.

### Immunoprecipitation experiments

Kelly cells (80-90% confluence) were transfected with 2 μg PTPRD cDNA or empty vector (pcDNA3.1-V5) in 100 mm dishes. After 24 h transfection, cells were incubated in a RIPA buffer (Sigma-Aldrich, Arklow) containing complete protease inhibitor mixture. The lysates were cleared by centrifugation for 20 min at 14,000 × g and then subjected to immunoprecipitation with either anti-V5 agarose affinity beads (Sigma-Aldrich) for co-IP experiments or 4 G10^® ^Platinum, anti-phosphotyrosine agarose conjugate (Millipore) for pan-tyrosine IP experiments, and then incubated at 4 C for 2 h. The beads were washed five times with RIPA buffer, incubated in laemmli sample buffer and heated to 95 C for five mins. Resultant samples were separated by SDS-PAGE and were analysed by western blotting.

### Western blotting

Cells were washed with PBS and lysed at 4 C for 20 min using RIPA buffer containg phosphatase and protease inhibitors. Lysates were centrifuged for 20 min at 14,000 × g and resultant protein extracts were separated by SDS-PAGE and blotted onto PVDF membranes. Antibodies used were as follows: anti-V5 (Invitrogen), anti-AURKA (Sigma), anti-MYCN (Santa Cruz) and anti-alpha tubulin (Abcam). Secondary antibodies were horseradish peroxidise-conjugated rabbit anti-mouse (Abcam) or goat anti-rabbit (Cell Signaling Technology). Reactive proteins were visualized by chemiluminescence with ECL (Thermo).

### PTPRD Interaction arrays

Purified PTPRD protein (99% pure, mass spectrum verified) (Clone ID 8613c5BCt10p1, protein sequence 1201-1495) was biotinylated and quantitated in duplicate using the Invitrogen FluoReporter^® ^Biotin Quantitation Assay Kit. The array control protein (biotinylated calmodulin kinase) supplied as part of the Invitrogen protein interaction kit was used to probe the Protoarray^® ^control protein microarray to validate assay conditions. Biotinylated PTPRD baits (two biological replicates) were then used to probe the Invitrogen ProtoArray^® ^Protein Microarray Kit for biotinylated proteins Version 4 in order to detect potential interaction partners. The results were analyzed using the Protoarray prospector^® ^package. The consensus hits (hits that are above the back ground signal in both the experiments) along with their Z-scores are listed in Table 1. A Z-Score indicates how far and in what direction the value of an individual data point in a population falls from the mean in units of standard deviations. ProtoArray^® ^Prospector collects all the signals from the proteins on a microarray, calculates the mean value and standard deviation, and then calculates a Z-Score corresponding to the signal from each feature.

### Cycloheximide half-life experiments

Kelly cells (80-90% confluence) were transfected with 2 μg PTPRD cDNA, empty vector (pcDNA3.1-V5) or PTPRD mutant (Q1481X) in 100 mm dishes. After 72 h transfection, cells were incubated in a RIPA buffer (Sigma-Aldrich, Arklow) containing complete protease inhibitor mixture. Cells were treated with 1 μl of a 20 mg/ml stock solution of cycloheximide (Sigma-Aldrich) 5 min prior to starting the indicated time course, and cells were collected at the indicated points. Quantification of western blots was performed by densitometry using GelEval (FrogDance Software). Normalization for loading differences was achieved by dividing the densitometry values for individual bands by the densitometry values for alpha-tubulin in the same lane.

## Abbreviations

AURKA: aurora kinase A; PTPRD: Protein Tyrosine Phosphatase Receptor Delta; IP: immunoprecipitation

## Competing interests

The authors declare that they have no competing interests.

## Authors' contributions

MM, LP, EL, NF, JR conceived and performed experiments and assisted with writing the manuscript. CAJ, NM, DM and RLS conceived experiments and assisted with writing the manuscript. BO, AF and TAC provided unique research materials. All authors read and approved the final manuscript

## Supplementary Material

Additional File 1 **Expression of PTPRD (A) mRNA and (B) protein following transfection of Kelly cells with increasing concentrations of *PTPRD *cDNA**. mRNA was extracted at 24 hours and qPCR was performed. Lysates were harvested at 48 h and subjected to SDS PAGE and western blot analysis with a monoclonal antibody to the V5 epitope tag or alpha tubulin. All experiments were performed in triplicate. Click here for file

Additional File 2 **(A) Expression of AURKA mRNA 48 hours post PTPRD expression**. Either 1 μg of PTPRD or empty vector (E.V.) were transfected into Kelly cells. mRNA was extracted at 48 hours and qPCR was performed. The figure is representative of four independent experiments and E.V. is set as 1.0. (B) Expression of MYCN mRNA 48 hours post PTPRD expression. Either 1 μg of PTPRD or empty vector (E.V.) were transfected into Kelly cells. mRNA was extracted at 48 hours and qPCR was performed. The figure is representative of four independent experiments and E.V. is set as 1.0. Click here for file
