# Supplementary figures and images for: Protein tyrosine phosphatase receptor delta acts as a neuroblastoma tumor suppressor by destabilizing the aurora kinase a oncogene
Source: Mol Cancer. 2012 Feb 5;11:6. doi: 10.1186/1476-4598-11-6 (PMC3395855; doi:10.1186/1476-4598-11-6)

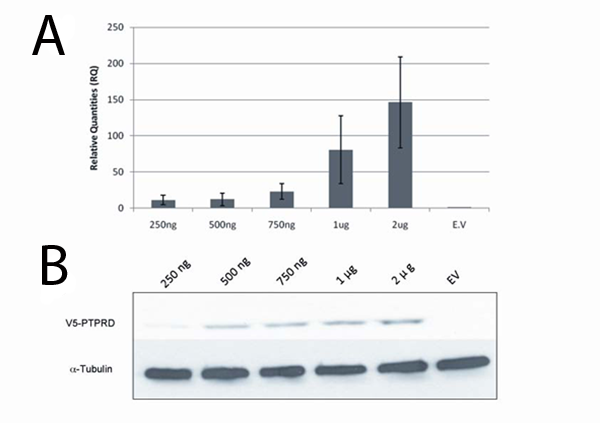

Supplement: Additional File 1 — Expression of PTPRD (A) mRNA and (B) protein following transfection of Kelly cells with increasing concentrations of PTPRD cDNA. mRNA was extracted at 24 hours and qPCR was performed. Lysates were harvested at 48 h and subjected to SDS PAGE and western blot analysis with a monoclonal antibody to the V5 epitope tag or alpha tubulin. All experiments were performed in triplicate. [file 1476-4598-11-6-S1.TIFF]

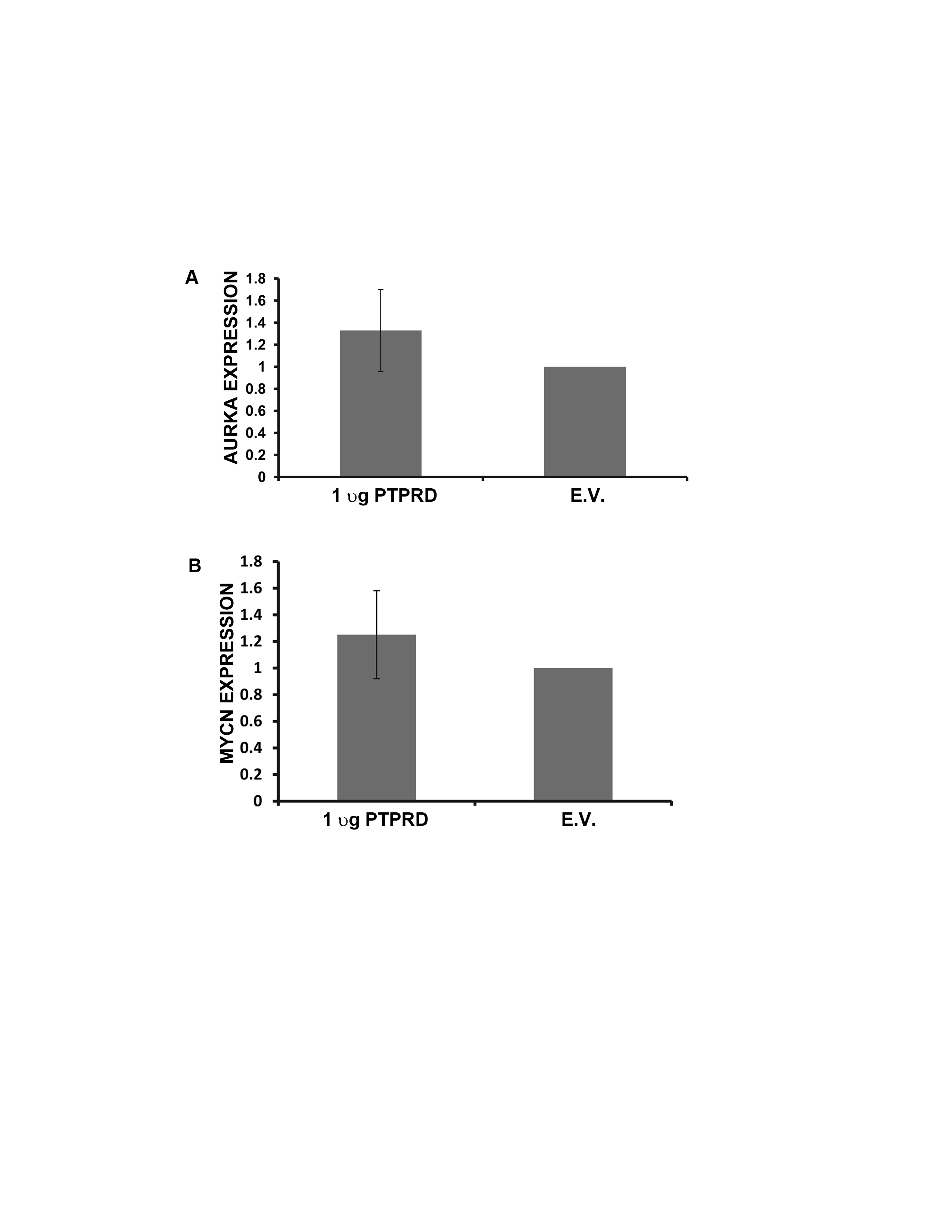

Supplement: Additional File 2 — (A) Expression of AURKA mRNA 48 hours post PTPRD expression. Either 1 μg of PTPRD or empty vector (E.V.) were transfected into Kelly cells. mRNA was extracted at 48 hours and qPCR was performed. The figure is representative of four independent experiments and E.V. is set as 1.0. (B) Expression of MYCN mRNA 48 hours post PTPRD expression. Either 1 μg of PTPRD or empty vector (E.V.) were transfected into Kelly cells. mRNA was extracted at 48 hours and qPCR was performed. The figure is representative of four independent experiments and E.V. is set as 1.0. [file 1476-4598-11-6-S2.TIFF]
